# The impact of D-cycloserine and sarcosine on in vivo frontal neural activity in a schizophrenia-like model

**DOI:** 10.1186/s12888-019-2306-1

**Published:** 2019-10-25

**Authors:** Lulu Yao, Zongliang Wang, Di Deng, Rongzhen Yan, Jun Ju, Qiang Zhou

**Affiliations:** 1 School of Chemical Biology and Biotechnology, Peking University Shenzhen Graduate School, Shenzhen, 518055 China; 2 State Key Laboratory of Chemical Oncogenomics, Key Laboratory of Chemical Genomics, Peking University Shenzhen Graduate School, Shenzhen, 518055 China

**Keywords:** NMDARs, Schizophrenia, NMDAR co-agonist, Calcium imaging, Single unit recording

## Abstract

**Background:**

N-methyl-D-aspartate receptor (NMDAR) hypofunction has been proposed to underlie the pathogenesis of schizophrenia. Specifically, reduced function of NMDARs leads to altered balance between excitation and inhibition which further drives neural network malfunctions. Clinical studies suggested that NMDAR modulators (glycine, D-serine, D-cycloserine and glycine transporter inhibitors) may be beneficial in treating schizophrenia patients. Preclinical evidence also suggested that these NMDAR modulators may enhance synaptic NMDAR function and synaptic plasticity in brain slices. However, an important issue that has not been addressed is whether these NMDAR modulators modulate neural activity/spiking in vivo.

**Methods:**

By using in vivo calcium imaging and single unit recording, we tested the effect of D-cycloserine, sarcosine (glycine transporter 1 inhibitor) and glycine, on schizophrenia-like model mice.

**Results:**

In vivo neural activity is significantly higher in the schizophrenia-like model mice, compared to control mice. D-cycloserine and sarcosine showed no significant effect on neural activity in the schizophrenia-like model mice. Glycine induced a large reduction in movement in home cage and reduced in vivo brain activity in control mice which prevented further analysis of its effect in schizophrenia-like model mice.

**Conclusions:**

We conclude that there is no significant impact of the tested NMDAR modulators on neural spiking in the schizophrenia-like model mice.

## Background

The glutamate hypofunction model of schizophrenia proposes that reduced function of glutamate receptors, especially NMDA subtypes on the inhibitory neurons leads to an imbalanced excitation and inhibition, and results in altered neural network functions (such as oscillations and working memory) [1–4]. These altered functions are proposed to drive the pathogenesis of schizophrenia and are likely contribute to on-going schizophrenia pathology. Thus, enhancing NMDARs may have therapeutic potentials in treating schizophrenia [5–8].

NMDAR, as a coincidence detector, its activation requires binding of glutamate, co-agonist (glycine or D-serine) and postsynaptic depolarization [8–11]. To avoid the excitotoxicity of direct activation of the NMDAR, targeting co-agonist/glycine binding site for enhancing NMDAR activation may be a good strategy for schizophrenia treatment [12–14]. The concentrations of co-agonist were reduced in the brains of schizophrenia patients [15–18], and administering NMDAR co-agonists (glycine, D-serine or D-cycloserine (DCS), etc) improved functions in schizophrenia patients in clinical trials [19, 20]. However, positive outcomes were not seen in some clinical trials [21–23], especially larger trials [24–26]. In addition to directly administering NMDAR co-agonists, another widely pursued approach is to elevate the endogenous glycine level by blocking its uptake using selective glycine transporter inhibitors, such as glycine transporter 1 (GlyT-1) inhibitors [27–29]. This approach takes advantage of the unique distribution of GlyT-1 in the brain. GlyT-1 plays a crucial role in regulating the availability of glycine near the vicinity of NMDARs and is expressed at both presynaptic and postsynaptic regions of glutamatergic synapses, as well as on the astrocytes [30–32]. GlyT-1 inhibitors have been recently tested in clinical trials aiming to treat negative symptoms in schizophrenia [28, 29, 33, 34]. Bitopertin, a GlyT-1 inhibitor developed by Hoffmann-La Roche, has reached phase III clinical trial for treating negative or positive symptoms in schizophrenia, but was halted due to lack of efficacy in improving negative symptoms which is the primary endpoint for these trials [35, 36]. Thus, the lack of success or at least inconsistent efficacy in treating schizophrenia by the NMDAR modulators has raised the question of whether enhancing NMDARs is the appropriate strategy in treating schizophrenia. Therefore, a careful analysis of the potential mechanisms and efficacy of these enhancers is critical for understanding why this approach has not met with success.

Clinical trials have focused on and aimed at improving functions or reducing symptoms in the schizophrenia patients. Although these are important readouts in determining the therapeutic values of a drug, it does not necessarily provide essential information on target engagement (i.e., whether enhanced activation of NMDARs has been achieved with drug on board) and whether this engagement translates to improved neural functions directly related to NMDAR activation, such as neural spiking. Elevating the level of co-agonist by exogenous glycine/D-serine or GlyT-1 inhibitor increased the activation of NMDARs in brain slices, and NMDA-induced neuronal spiking in vivo [37–41]. The expression of LTP is enhanced in the presence of glycine in vitro [42, 43], but whether glycine/D-serine/DCS is sufficient to increase neural spiking has not been systematically examined.

Neuregulin1 (NRG1) is a transmembrane protein with an epidermal growth like-domain, and important for neuronal developmental process and functions [44]. NRG1and its receptor ErbB4 are susceptibility genes in schizophrenia [45, 46]. In the postmortem brains of schizophrenia, expression of NRG1 mRNA and protein was upregulated [47, 48]. NRG1 signaling could be mediated via canonical and non-canonical pathways, with the former requires NRG1 binding to ErbB4 receptor, and the latter not [44]. We used an anti-Neuregulin1 (anti-Nrg1) antibody to induce schizophrenia-like phenotypes, including hyperlocomotion and impaired prepulse inhibition [49, 50]. Injection of this antibody stabilized the full length Nrg1 and caused it to accumulate, based on in vitro evidence of robust increase of full length NRG1 in Nrg1-expressing 293 cells treated with anti-NRG1 antibodies. It was suggested that anti-Nrg1 antibodies likely activate ErbB4-independent signaling and resulted in increased phosphorylation of cofilin [50]. This signaling pathway is similar to that reported with overexpression of NRG1 [51]. The advantage of this anti-Nrg1 antibody model over the more widely used genetic, pharmacological or lesion models is that schizophrenia-like phenotypes can be induced in the adult mice (Ju et al., 2019) and thus offers one rare opportunity to study late/adult-onset schizophrenia model. At least 20% of schizophrenia cases are of late/adult onset after the age of 40 years [52]. We tested the effect of NMDAR modulators on this schizophrenia-like model.

In the current study, we examined whether glycine, DCS or sarcosine significantly affects neuronal spiking in schizophrenia-like model mice. We have used both in vivo Ca^2+^ imaging and in vivo single unit recording. Somatic Ca^2+^ responses allowed us to measure neuronal spiking activity in certain types of neurons (depend on the promoter used), while in vivo single unit recording allows measurement of changes in both single spikes and burst spikes in response to administration of NMDAR modulators. We have observed an increased neural activity in the schizophrenia-like model mice induced by anti-Nrg1 antibodies, but did not find any significant change in neural activity using both measurements after administration of NMDAR modulators in either control or schizophrenia-like model mice.

## Methods

### Animals

C57BL/6 wildtype mice were purchased from Guangdong Medical Laboratory Animal Center. All animal experiments were performed in accordance with the ARRIVE guidelines on the Care and Use of Experimental Animals, approved by the Peking University Shenzhen Graduate School Animal Care and Use Committee. To establish a schizophrenia-like model, anti-Nrg1 antibody (a gift from Genentech) was intraperitoneally injected into 8 weeks old male mice (20 mg/kg,) three times within a 2 week period, while control mice were intraperitoneally administered with anti-Ragweed (anti-Rag) (20 mg/kg) [49, 50] . Mice were randomly divided into the two groups. All efforts were made to reduce the number of animals used and to minimize animal suffering during the experiment. We anesthetized the mice with isoflurane inhalation or intraperitoneal injection of phenobarbital sodium (100 mg/kg).

### Two-photon imaging

#### Surgery for two-photon imaging

Mice of 7~8-weeks of age were anaesthetized by intraperitoneal injection (100 mg/kg) of phenobarbital sodium. The fur over the injection site was shaved and an incision was made, and a small hole was drilled. A glass pipette was advanced towards the imaging area to at a 20° angel to minimize tissue disturbance. Each mouse was injected with virus (under either CAG, CaMKII or Synapsin) coding GCaMP6s (Penn Vector Core) in the frontal association cortex (coordinates: Bregma + 2.5–2.8 mm, midline: ± 1.0 mm, ventral: 0.2~0.3 mm) [53]. The skin was then sutured to allow viral expression for 14 days in the home cage prior to two-photon imaging. During this viral expression period, anti-Nrg1 antibody or anti anti-Rag antibody was injected into mice i.p.

**Fig. 1:**
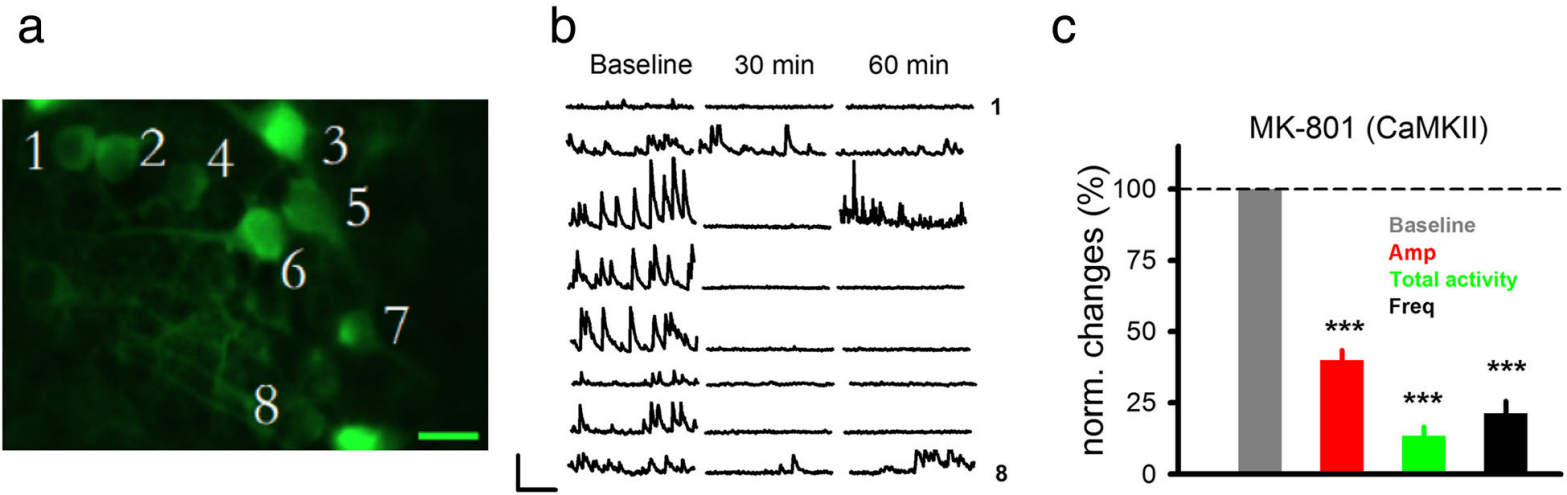
Spontaneous in vivo Ca^2+^ responses in the frontal cortex neurons and their sensitivity to NMDAR antagonist MK-801. **a** Sample image of neurons expressing CaMKII-GCaMP6s virus (green) in the frontal cortex. Scale bar, 10 μm. **b** Sample traces of spontaneous Ca^2+^ response before (baseline), 30 and 60 min after injection of MK-801 (0.3 mg/kg, i.p). Scale bars, 1 min; 4%ΔF/F_0_. **c** Quantification of Ca^2+^ responses and the effect of MK-801, 30 min after MK-801 injection. Dotted line indicates baseline levels, normalized to 100%. Amp, amplitude; Freq, frequency

Calcium responses in the somata of layer II/III FrA neurons were collected since FrA has significant relevance to schizophrenia pathology and is more accessible for in vivo two photon imaging than medial prefrontal cortex [53, 54] (Fig. 1a). CAG is a pan-neuronal promoter that does not distinguish between excitatory and inhibitory neurons, while CaMKII allows selective expression of GCaMP6s in the excitatory neurons. By using these two promoters, we can evaluate the impact of NMDAR modulators on either the entire neuronal population or excitatory neurons selectively.

Under intraperitoneal injection of phenobarbital sodium (100 mg/kg), the fur over most of the scalp, skin and connective tissues over the skull were removed. The mouse head was held by placing two steel bars centered on the region to be imaged. A thick layer of freshly mixed dental cement was applied to keep the bars in place. Mice were put back to their home cages and imaging was performed on the next day.

#### Imaging and analysis

High-speed micro-drill was used to thin (20 μm) circular area of skull [55]. A 25× objective (N.A. 1.05; 2X digital zoom) was used to image in time-lapse mode with the image size of 512 × 512 pixels and the temporal resolution is 1.109 s per frame (about 0.9 Hz). Imaging was performed for 2~3 times to establish a basal level of calcium responses prior to administration of MK-801 (0.3 mg/kg) in wild type mice, DCS (30 mg/kg, i.p) or sarcosine (0.3 g/kg or 1 g/kg, i.p) in schizophrenia-model mice. About 30 min after dosing, images were taken for 1 h with an interval of 10 min. The selection of this imaging time frame was based on the published results on the time taken for DCS and glycine to rise significantly after dosing. For DCS, it was reported that the brain concentration of DCS increased to 0.68 ± 0.28 to 0.86 ± 0.37 μ mol/g at 15 to 60 min following 320 mg/kg i.p [56]. For sarcosine, the concentration in the serum was about 18 to 25 μ mol/L at 30 to 120 min following 2 g/d oral intake [57], and the availability of glycine increased by 50 to 100% at 20 to 60 min following i.p [58]. On the next day, a different drug was injected if the mouse was in good condition (no serious inflammation, no bleeding in the thinned-skull area); otherwise, the mouse was sacrificed. After completion of experiments, mice were euthanized with minimum pain using carbon dioxide.

Regions-of-interest (ROIs) were manually selected in Image J software (NIH) to include all neuronal somata that appeared in all time-lapse image sets. We reported time series as ΔF/F = (F-F_0_)/ F_0_, where F is the fluorescence signal after background subtraction, and F_0_ is the mean of minimum fluorescence signal during a baseline period. The threshold for determining calcium signal was calculated as three times the standard deviation (SD). To calculate the integrated area/total activity, the area under Ca^2+^ responses within 1 min was integrated using Origin 7.0 software. For experiments using CAG promoter and CaMKII promoter (data shown in Figs. 3 and 4), neurons with their nucleus filled with fluorescence were not included in the analysis. For the MK-801 and sarcosine experiments (data shown in in Figs. 1 and 5), about 70% of neurons had nucleus filled with fluorescence. However, we found that all imaged neurons (whether they were filled or not) respond in the same way to MK-801 or sarcosine injection. Thus, all neurons were included in the analysis for these two experiments.

### In vivo single unit recording

#### Surgery and recordings

Wild type mice, mice injected with anti-Nrg1 antibody or anti-Rag (control) antibody were anesthetized with isoflurane and secured in a stereotaxic frame. As previously described [59], a multi-wire electrode set was unilaterally implanted aiming at medial prefrontal cortex (mPFC) with the following coordinates: 1.94 mm anterior to Bregma; 0.4 mm lateral to midline; 2.65 mm ventral from the cortical surface. After surgery, mice were allowed to recovery for 7 to 14 days. Neuronal activity was digitized at 30 kHz and band-pass filtered from 250 Hz to 5 kHz. During recording, 10 min baseline spike rate was collected before drug injection (Saline or DCS (30 mg/kg) or MK-801 (0.3 mg/kg), all i.p) and recorded for another 60 to 90 min after drug injection. Mice were euthanized with carbon dioxide after completion of experiments.

#### Spike sorting and analysis

Analysis was based on methods published previously [59]. Briefly, waveforms of all recorded neurons were aligned using an offline-sorter software (Plexon). The principal component analysis (PCA) was then used to calculate the principal component score of the unsorted neuronal waveforms, and scores were plotted in a three-dimensional principal component spaces. Burst spikes are based on the following criteria: (1) inter spike interval less than 15 ms; (2) spike count in one burst larger than 2; (3) inter-burst interval longer than 100 ms. Relative change in spike rate (SR) is calculated as: Mean firing rate (after drug injection) /Mean firing rate (before drug injection) × 100 (%).

### Open field test

Measurement of locomotion was based on method described previously [49]. In brief, mice were placed in the testing room for 1–2 h to be familiar with the environment prior to testing. Total distance traveled and time spent in the center area during a 60 min period was recorded using ANY-maze software (Global Biotech Inc.). D-cycloserine (30 mg/kg) or glycine (1.6 g/kg) was injected (i.p) after 30 min in the open field.

### Statistics

In general, normalized values were calculated as percentage change from baseline. Data can be fitted with a Gaussian distribution and were analyzed using two-way ANOVA with Bonferroni post-tests, unpaired t-test or paired t-test, and presented as mean ± SEM using Graph prism. Significant levels were set at *P* < 0.05 *, *P* < 0.01 **, *P* < 0.001, ***.

## Results

In vivo Ca^2+^ imaging and in vivo single unit recording were used to examine potential modulation of neuronal activity by NMDAR modulators in both schizophrenia-like model and control mice. These two approaches are complementary to each other and provide a comprehensive view with both spatial and temporal resolution of in vivo neural activity, as well as contribution of excitatory neurons.

### In vivo Ca^2+^ signals and the effects of NMDAR inhibitor MK-801

To obtain in vivo Ca^2+^ signals, virus (under either CAG or CaMKII promoter) coding GCaMP6s was injected into mouse frontal association cortex (FrA). We focused on the Ca^2+^ responses in the somata of layer II/III FrA neurons since FrA has significant relevance to schizophrenia pathology and is more accessible for in vivo two photon imaging than medial prefrontal cortex [53, 54] (Fig. 1a).

We recorded spontaneous Ca^2+^ activity in the frame scan mode and found robust elevation in fluorescence indicating elevation in the intracellular Ca^2+^ concentrations (Fig. 1b, as an example). Somatic Ca^2+^ responses reflect spiking activity most likely mediated by opening of voltage-gated Ca^2+^ channels on the somata or diffusion of Ca^2+^ from nearby dendrites upon depolarization [60]. Since the goal of the study is to determine whether NMDAR modulators may affect in vivo neural activity in schizophrenia model mice, we first wanted to determine whether activation of NMDARs have significant contribution to the recorded Ca^2+^ signals. To do so, we tested the effect of selective NMDAR antagonists MK-801.

We found a significant reduction in the Ca^2+^ signals starting 30 min after MK-801 injection (i.p. 0.3 mg/kg; Fig. 1b and c; 84 somas/3 mice, Amp = 39.89 ± 3.48%; Total activity = 13.36 ± 3.21%; Freq = 21.14 ± 4.38%; all normalized to baseline (pre-MK-801) level (100%); *P* < 0.001). The significant reduction in Ca^2+^ responses indicated that NMDARs play a critical role in the observed Ca^2+^ responses. Previous studies have shown that MK-801 injection resulted in a significant reduction in the burst spikes in the prefrontal neurons [61], suggesting that our Ca^2+^ responses could largely be mediated by burst spikes (*see* *Discussion*).

**Fig. 2:**
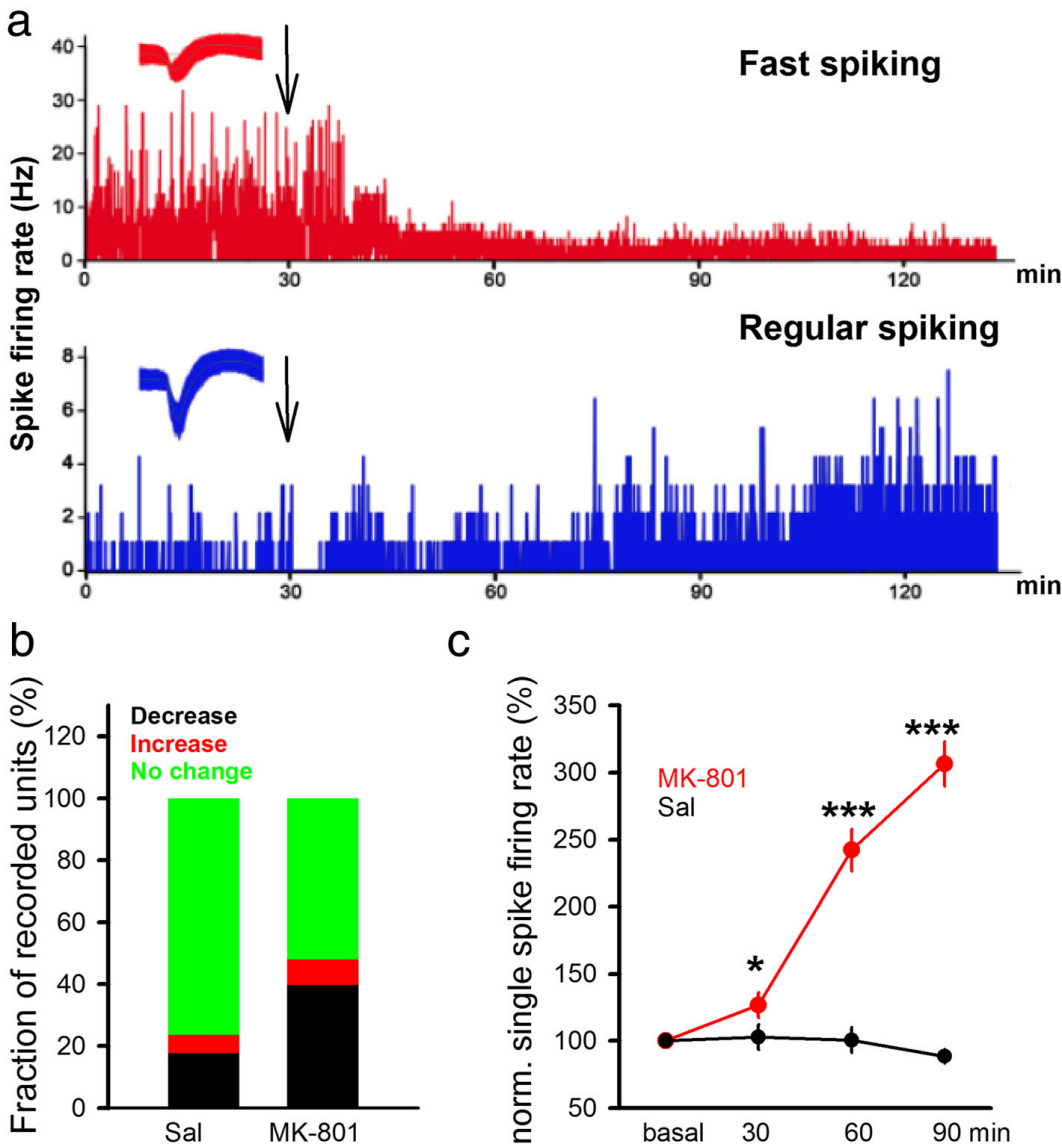
In vivo recording demonstrated differential changes in neural activity after injection of MK-801. **a** Sample recording of in vivo single unit recording of neural activity in the mPFC (Upper). In a fast-spiking neuron (basal spike frequency > 10 spike/sec), injection (i.p) of MK-801 (0.3 mg/kg, arrow) led to a reduction in neural spiking over time. (Lower) In a regular spiking neuron, MK-801 injection led to an increase in neural activity over time. Sample spike waveforms were shown in the inserts. **b** Fraction of recorded units showing no change, increase and decrease in burst spike rate in the saline (Sal) and MK-801 injection group. Burst spike rate were not altered in most neurons in the Sal group, while nearly twice as many unit showed significant decrease in burst spike rate in the MK-801 group. The group showing decrease represents the average burst firing rate following 30 to 60 min after administration of MK-801. Decrease is defined as reduced by 20% or more, increase defined as increase by 20% or over, and no change defined as values between 80 and 120%, all compared to baseline. **c** Normalized changes in single spike rates before, 30, 60 and 90 min after MK-801 injection. Significant increase was seen starting 30 min after MK-801 injection

Previous works using in vivo single unit recording showed a reduction in spike rate in the putative inhibitory neurons and a concomitant increase in spike rate of excitatory neurons after systemic injection of MK-801 [61, 62]. After i.p injection of MK-801 (0.3 mg/kg) in wild type mice, we found a reduction in spike rate in the fast-spiking neurons (likely inhibitory neurons) and an enhancement in firing rate in the regular spiking neurons (likely excitatory neurons) (Fig. 2a). A higher percentage of units showing reduction in burst spike (Fig. 2b, saline, 96 units/7mice; MK-801, 71 units/5mice, saline = 17.64%, MK-801 = 39.58%) was seen with a significant increase in single spike frequency starting 30 min after MK-801 injection (Fig.2c, saline =102.81 ± 9.29%, MK-801 = 126.50 ± 9.37%; unpaired t-test, *P* < 0.05). Taken together, our findings are consistent with previous studies regarding changes in spiking after administration of NMDAR blockers, and further suggest that the recorded Ca^2+^ signals most likely reflect burst spike rather than single spikes (*see* *Discussion*) [61, 63].

### Elevated neural activity in the frontal cortex of schizophrenia-like model mice

After confirming that our methods (imaging and recording) can readily detect changes in neural activity caused by NMDAR blocker, and consistent with published results, we set to examine the impact of NMDAR modulators on neural activity in schizophrenia model. First, we examined whether the general activity level is altered in our schizophrenia model mice. Previous studies showed that injection of anti- Nrg1 antibodies led to the appearance of schizophrenia-like phenotypes in mice [49, 50], which is likely caused by elevated Nrg1 signaling [50]. In addition, enhanced synaptic transmission onto excitatory neurons was also seen in the anti-Nrg1 mice [49], consistent with other reports of hyperactivity in mice with NMDAR hypofunction in the inhibitory neurons [2]. Thus, we first examined in vivo cortical activity in the anti-Nrg1 mice.

**Fig. 3:**
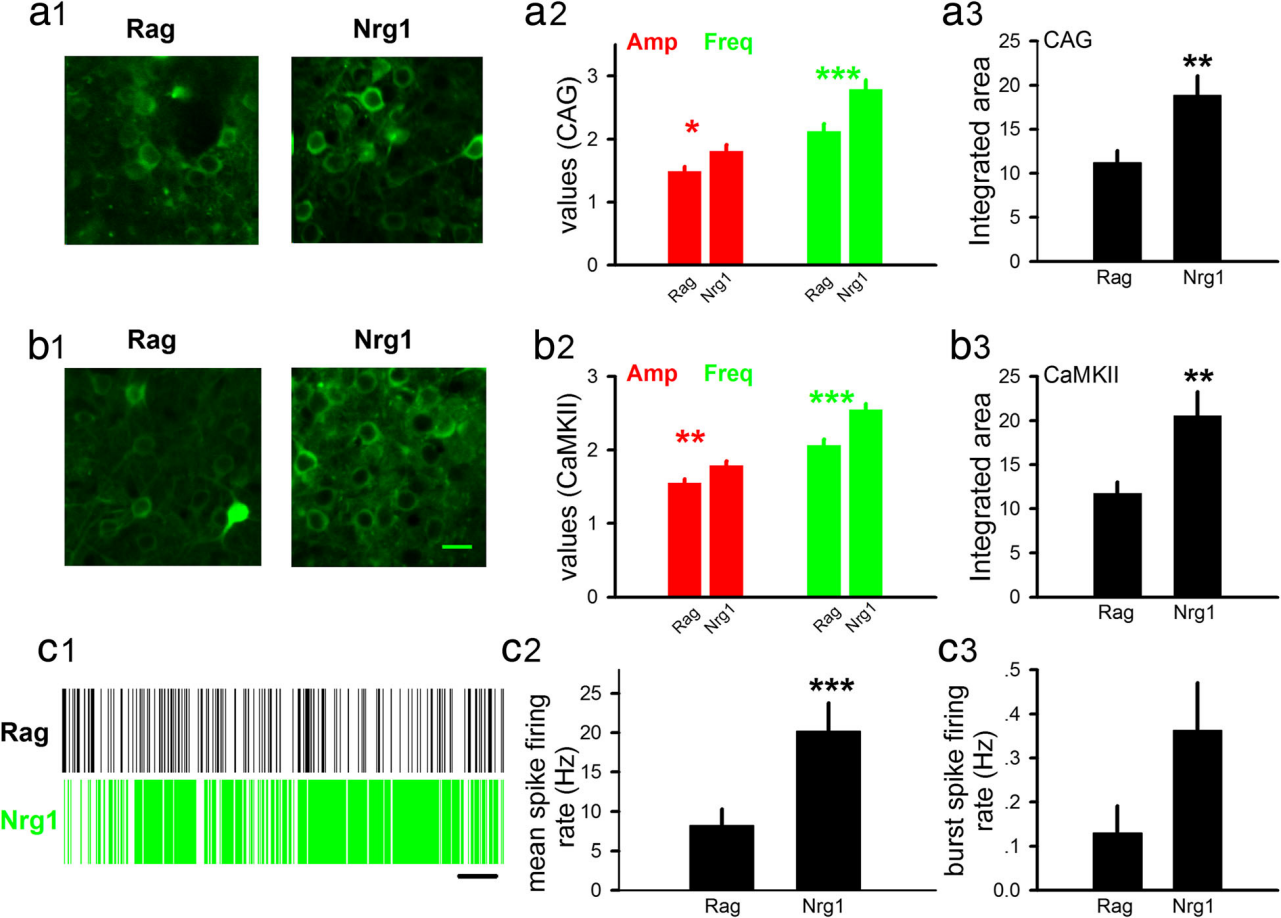
Elevated basal neuronal activity in mice injected with anti-Nrg1 antibodies. **a**. Neural activity measured using CAG-GCaMP6s virus in FrA neurons. (A1) Sample images of neurons expressing CAG-GCaMP6s virus in the FrA, in mice injected with either anti-Rag antibody (left, Rag) or anti-Nrg1 antibody (right, Nrg1). (A2) Individual spontaneous Ca^2+^ response showed increase in amplitude (amplitude of ΔF/F), and frequency (number of events per min), in mice injected with anti-Nrg1 antibodies. (A3) Total Ca^2+^ activity (integrated area of all events within 1 min) was also elevated in anti-Nrg1 mice. Scale bars, 10 μm. **b**. Neuronal activity in the excitatory neurons measured using CaMKII-GCaMP6s virus in the FrA. (B1) Sample images of neurons expressing CaMKII-GCaMP6s virus in the anti-Rag (left) and anti-Nrg1 mice (right). (B2) Amplitude and frequency of individual spontaneous Ca^2+^ responses were significant higher in the anti-Nrg1 mice, compared to the anti-Rag mice. (B3) Total Ca^2+^ activity level was higher in the anti-Nrg1 mice compared to the anti-Rag control mice. Scale bars, 10 μm. **c**. In vivo single unit recording. (C1) Sample recording in an anti-Rag (upper) or anti-Nrg1 mouse (lower). Each vertical line represents a single spike. Scale bar, 50 s. (C2) Significantly higher mean spike rate was seen in the anti-Nrg1 mice, compared to the anti-Rag mice. (C3) Burst spike rate was not significantly different between the anti-Nrg1 and anti-Rag mice. *, *P* < 0.05; **, *P* < 0.01, ***, *P* < 0.001

In neurons expressing CAG-GCaMP6s, we found a significant increase in the spontaneous Ca^2+^ responses in anti-Nrg1 mice, compared to mice injected with anti-Rag antibodies (Fig. 3A1). This increase was seen in the amplitude and frequency of individual Ca^2+^ responses (Fig. 3A2). Total neural activity was quantified by integrating Ca^2+^ signals and a significant increase in the anti-Nrg1 mice was found (Fig. 3a; 113 somas/5 mice (Rag), 116 somas/6 mice (Nrg1); unpaired t-test, *P* < 0.01), indicating an increase in neural activity. We further examined whether this increased neural activity occurred in the excitatory neurons by monitoring Ca^2+^ responses in neurons expressing CaMKII-GCaMP6s (Fig. 3B1). A significant increase in the amplitude and frequency of Ca^2+^ responses, and the total activity (Fig. 3B2&3B3; Amp = 1.55 ± 0.05 (Rag), 1.78 ± 0.05 (Nrg1), unpaired t-test, *P* < 0.01; Freq = 2.06 ± 0.07 (Rag), 2.55 ± 0.08 (Nrg1), unpaired t-test, *P* < 0.001; 157 somas/4 mice (Rag), 228 somas/8 mice (Nrg1), Rag = 11.75 ± 1.28, Nrg1 = 20.58 ± 2.71; unpaired t-test, *P* < 0.01) was found, indicating elevated neuronal activity occurs in the excitatory neurons.

To confirm the above findings using an independent measurement, we recorded neural activity using in vivo single unit recording. We found a significant elevation in the mean firing rates (mostly single spikes) (Fig. 3C2; 88 units/3 mice (Rag), 78 units/3 mice (Nrg1); unpaired t-test, *P* < 0.001). Burst spike rate was not altered (Fig. 3C3; 41 units/3 mice (Rag); 55 units/3 mice (Nrg1)). Note the large increase in mean firing rate in the anti-Nrg1 mice (250%, 8 ± 2.10 Hz (Rag), 20 ± 3.57 Hz (Nrg1)). Thus, the elevated neural activity appears to be mostly associated with an increase in single spikes.

### D-cycloserine did not affect neuronal activity in the schizophrenia-like model mice

**Fig. 4:**
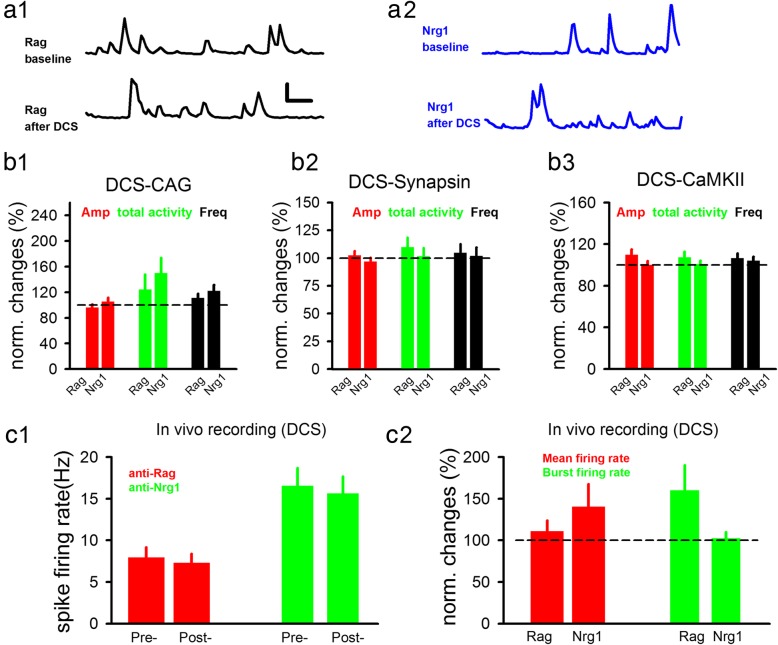
Effect of DCS injection on neural activity in schizophrenia-like model mice in vivo. **a**. Sample traces of Ca^2+^ responses before and after injection of DCS (i.p, 30 mg/kg) in either anti-Rag mice (A1) or anti-Nrg1 mice (A2) expressing CAG promoter virus. Scale bars, 0.2 min; 400% ΔF/F_0_. **b**. Spontaneous Ca^2+^ responses were not altered by DCS injection, as measured by Ca^2+^ activity reported using CAG-GCaMP6s (B1), DCS-Synapsin (B2) or CaMKII-GCaMP6s (B3). Dotted line indicates baseline levels, normalized to 100%. **c**. No significant change in either mean spike rate (C1, anti-Rag, 88 units/3 mice; anti-Nrg1, 78 units/3 mice) or burst spike rate (C2, anti-Rag, 41 units/3 mice; anti-Nrg1, 55 units/3 mice)) after DCS injection in anti-Nrg1 mice. Dotted line indicates baseline levels, normalized to 100%

As stated in the Introduction, although it has been shown that glycine/D-serine can effectively enhance synaptic NMDAR function/activity in mice [38, 39], a critical question regarding the in vivo efficacy of exogenous co-agonist is whether this enhanced NMDAR function necessarily translates into change in in vivo spiking activity. Since DCS/glycine/GlyT-1 inhibitors have shown certain efficacy in schizophrenia patients and model mice, we expected that they do so by modulating neural activity. We examined Ca^2+^ responses before and after injection of DCS (30 mg/kg, i.p). We have observed hyperlocomotion in the anti-Nrg1 mice which was not significantly affected by DCS injection (Additional file 1: Figure S1; 8 mice (Rag), 8 mice (Nrg1)). Compared to anti-Rag mice, no significant effect of DCS was found on spontaneous Ca^2+^ responses in anti-Nrg1 mice (Fig. 4a, b; raw data shown in Additional file 2: Figure S2), in neurons expressing either CAG-GCaMP6s (Fig. 4B1 & Additional file 2: Figure S2; 77 somas/3 mice (Rag), 71 somas/4 mice (Nrg1)), Synapsin-GCaMP6s (Fig. 4B2; 83 somas/5 mice (Rag), 139 somas/4 mice (Nrg1)) or CaMKII- GCaMP6s virus (Fig. 4B3 & Additional file 2: Figure S2 120 somas/4 mice (Rag), 129 somas/5 mice (Nrg1)). As shown in Additional file 2: Figure S2, the increased basal neuronal activity in anti-Nrg1 mice was still observed, consistent with the Fig. 3. This conclusion is further supported by the absence of changes in in vivo spike frequency after DCS injection in either anti-Nrg1 or anti-Rag mice, on either mean spike rate or burst spike rate (Fig. 4c; 3 mice (Rag), 3 (Nrg1)).

### Glycine transporter inhibition does not affect neural activity

**Fig. 5:**
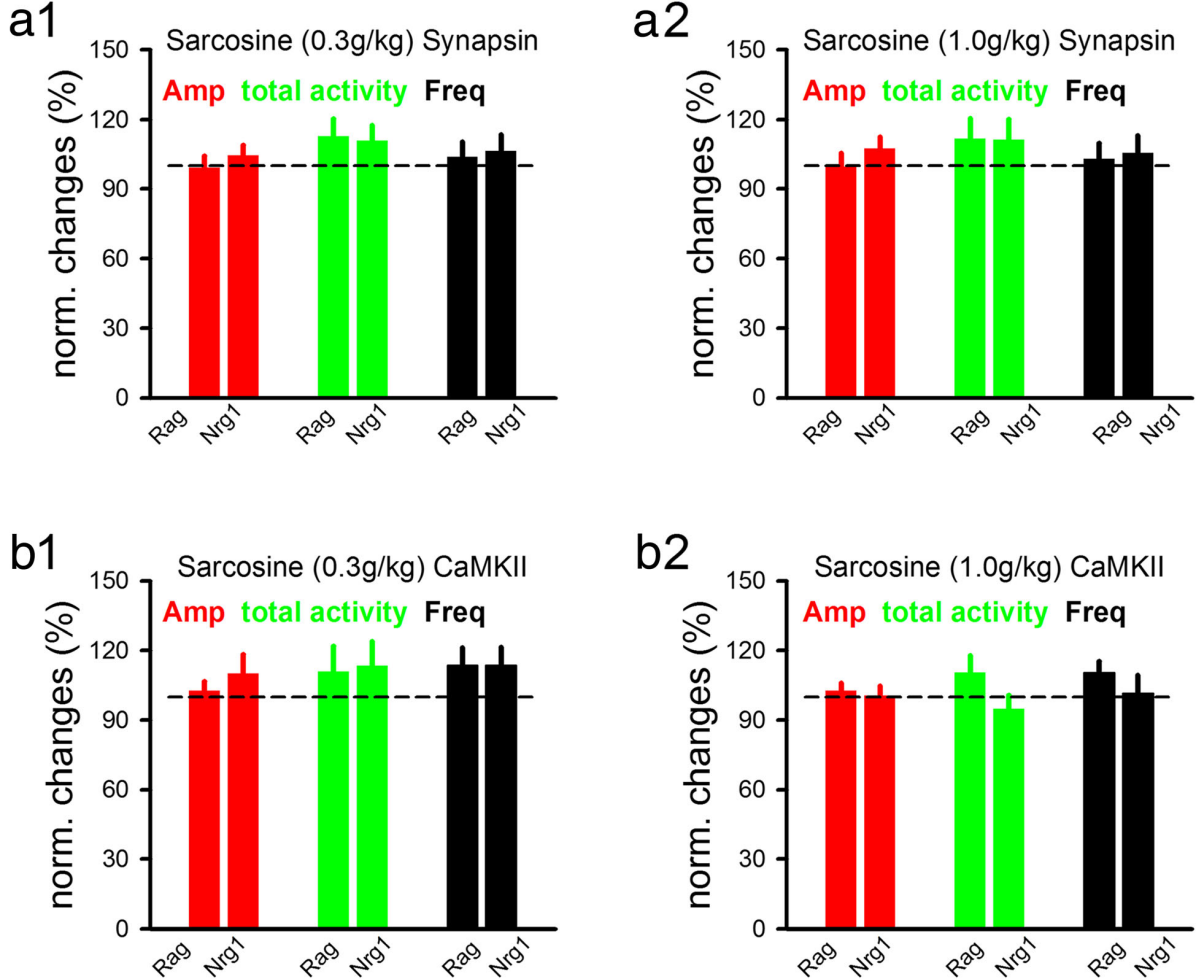
Effect of sarcosine on neural activity schizophrenia-like model mice. **a**. In neurons expressing synapsin-GCaMP6s virus, i. p. injection of sarcosine (A1; 0.3 g/kg; Rag, 104 somas/5 mice; Nrg1, 119 somas/4 mice) (A2; 1 g/kg; Rag, 94 somas/5 mice; Nrg1, 124 somas/4 mice) did not significantly change the amplitude, frequency or total activity of spontaneous Ca^2+^ responses, in either anti-Nrg1 or anti-Rag mice. Dotted line indicates baseline levels, normalized to 100%. **b**. In neurons expressing CaMKII-GCaMP6s virus, injection sarcosine (B1; 0.3 g/kg; Rag, 116 somas/4 mice; Nrg1, 60 somas/3 mice) (B2; 1 g/kg; Rag, 124 somas/5 mice; Nrg1, 85 somas/5 mice) did not significantly change the amplitude, frequency or total activity of Ca^2+^ responses, in either anti-Nrg1 or anti-Rag mice. Dotted line indicates baseline levels, normalized to 100%

Previous evidence showed that endogenous glycine concentration can be effectively enhanced by inhibiting its uptake with GlyT-1 inhibitors [64, 65]. Numerous GlyT-1 inhibitors with excellent drug-like properties have been tested in vivo [41, 66–69]. Here, we chose to use sarcosine which has been used widely in preclinical research [70, 71]. Two doses of sarcosine (0.3 g/kg and 1 g/kg, i.p) were used to examine Ca^2+^ responses in neurons expressing either synapsin-GCaMP6s (Fig. 5a, raw data shown in Additional file 3: Figure S3A) or CaMKII-GCaMP6s (Fig. 5b, raw data shown in Additional file 3: Figure S3B). We did not find any significant changes in Ca^2+^ responses, in either virus-infected neurons, for either dose, in either anti-Nrg1 or anti-Rag mice. These results suggest that enhancing the endogenous glycine level by inhibiting its uptake does not significantly alter neural activity.

In addition, we tested the effects of in vivo administration of glycine (1.6 g/kg, i.p) [72, 73]. We found a large reduction in the movement of control (anti-Rag) mice in their home cages after injection with glycine (data not shown). When locomotion was analyzed in the open field, we did not find that glycine injection caused significant changes to locomotion in either anti-Nrg-1 or anti-Rag mice (Sup. Additional file 4 Figure S4A). In addition, a significant reduction in the spontaneous spiking was seen from in vivo recording in the home cage (Additional file 4: Figure S4B-C). Since brain activity level is influenced by the locomotion level in the free-moving mice we studied, this reduced locomotion may interfere with the interpretation of glycine effects on NMDAR-dependent modulation of brain activity. Thus, we did not examine the effect of glycine injection on in vivo neuronal activity any further.

## Discussion

In this study, we addressed a simple question: does D-cycloserine or sarcosine alter neuronal spiking in the frontal cortex of schizophrenia-like model mice? By using combined in vivo two-photon time lapse Ca^2+^ imaging and in vivo single-unit recording, we found that spontaneous neuronal activity is significantly increased in schizophrenia-like model, but unaltered by administration of the above compounds, suggesting no major effects of the above NMDAR enhancers on neural spike activity in vivo in schizophrenia-like model mice.

### Technical considerations for what the signals represent

The majority of neural spiking measured in this study, either directly using in vivo recording or inferred from Ca^2+^ responses, come from excitatory neurons, as reflected by sampling bias of the recording electrodes, spike width and frequency of the recorded spikes, and promoter used to express GCaMP6s. Neuronal activity measured using Ca^2+^ imaging showed a huge reduction after MK-801 injection, while burst spike rate decreased and single spike rate significantly increased as measured using in vivo recording (Figs. 1, 2). Based on the comparison between Ca^2+^ imaging and in vivo recording, the Ca^2+^ activity we observed in the frontal neurons are likely mediated by burst spikes in neurons. This conclusion is consistent with the sensitivity of GCaMP6s shown in previous studies [63]. This technical aspect is important since it indicates that Ca^2+^ signals in our results mostly reflect synaptic inputs rather than random spiking of neurons (we imaged cell somas under resting/no stimulation condition), and hence more likely reflect changes in input-driven functions of these neurons. From in vivo recordings, we also obtained information on the spontaneous spiking (mostly single spikes), which likely reflects random spiking rather than input-driven neuronal activity. Random spiking could in some ways be viewed as noise rather than signals, or signals reflecting the internal state. Due to the high spike rate there is a possibility that some spikes occur close enough to each other temporally to trigger significant Ca^2+^ responses (i.e., they appear as burst spikes).

### Changes of neural activity in schizophrenia-like model mice

Previous works showed that administration of anti-Nrg1 antibodies induced schizophrenia-like behavioral phenotypes (including hyper-locomotion and impaired pre-pulse inhibition of startle) and altered synaptic transmission in adult mice [49, 50]. Compared to the more established models of schizophrenia in the literature, being genetic, pharmacological or lesion, our model of using anti-Nrg1 antibodies is fairly new and less established, regarding the characterization of behavioral phenotypes, pathophysiology, responses to drugs/treatment (such as antipsychotics) and the developmental time course of the landmark events. On other hand, one major advantage of our model is that the onset of schizophrenia can be controlled by the timing of antibody injection so that this is one of rare model to study late/adult onset schizophrenia (Ju et al., 2019).

Consistent with prior studies showing hyperactivity in schizophrenia-like models [74, 75], we found elevated in vivo activity, both in Ca^2+^ response and single spike rate. The increase in total activity revealed by Ca^2+^ responses is rather small (Fig. 3A3 & B3), but the increase in in vivo spike rate is fairly large (Fig. 3c). These differences are consistent with the majority of Ca^2+^ responses mediated by burst spikes, and also indicate that increase in single spike (random spiking) is a major electrophysiological alteration in schizophrenia-like models.

We found that injection of MK-801 led to an initial reduction in the spiking of presumed fast-spiking neurons followed by delayed increased spiking in the presumed excitatory neurons in the mPFC, consistent with those from previous studies [61, 62]. These results are also consistent with increased activity in anterior cingulate cortex in an acute MK-801 treatment model of schizophrenia using two-photon imaging [76]. Intriguingly, Ranson (2019) reported reduced activity in excitatory neurons in V1 in the same model. This reduction may be explained by suppression of V1 activity by anterior cingulate cortex inputs in this model, or regional differences in the distribution of NMDARs. It was well established that the distribution of NMDAR subunits is very strongly dependent on the specific brain regions [8].

In addition to the general level of neural activity, it is widely accepted that the synchronization of neural spiking either in the same brain region or between different brain regions is significantly altered in schizophrenia patients and mice [77–80]. Altered gamma oscillations have been reported, together with deficits in working memory [81–83]. Hippocampal-prefrontal synchrony during working memory task is impaired in a schizophrenia model [84], and deficits in frontal cortical gamma-band synchrony may contribute to the impaired cognitive control in schizophrenia [85]. Our current dataset does not have sufficient temporal resolution to perform such an analysis, but exploring whether synchrony of spiking is altered in our model mice and the potential impact of NMDAR modulators is of interest and importance in future studies.

### Potential explanations for the lack of effectiveness of NMDAR co-agonists on neuronal activity

There are a few potential possibilities for why this approach did not alter in vivo neuronal activity in schizophrenia-like model mice in this study: (1) Hypofunction of NMDARs in schizophrenia, especially the hypofunction of NMDARs on the inhibitory neurons [86, 87], which is supported by from both postmortem findings and preclinical studies using mice with reduced NMDAR expression in the inhibitory neurons [88–90]. However, if the density of NMDAR in inhibitory neurons is so low that the enhancement provided with NMDAR co-agonists may not be sufficient to increase spiking. This scenario is consistent with prior findings of enhanced synaptic NMDAR function by these modulators [11, 38, 65] but no impact on spiking in this study. Furthermore, the loss of inhibitory neurons reported in schizophrenia patients and model animals may have additional contribution to this lack of efficacy in increasing neuronal spiking [91–95]. In our schizophrenia-like model mice, it is unclear whether cell loss and NMDAR hypofunction occurs, although there is evidence for reduced NMDAR activation by NRG1 stimulation in the PFC of both human schizophrenia patients and rodent models [96, 97] . In the future studies, it is necessary to confirm the NMDAR hypofunction and cell loss in our model. (2) Saturation of the strychnine-insensitive glycine binding site on NMDARs of the co-agonists. To boost NMDAR activation by co-agonists, the binding sites need not to be saturated. Our results suggest that the binding sites might be saturated, consistent with prior in vivo study [98], although there are some evidence indicating the glycine binding site not saturated [40, 41]. However, whether the binding site is saturated needs to be determined in vivo since microenvironment might be altered in the brain slices [99]. Furthermore, whether glycine site is saturated in the schizophrenia brain is unknown. Since we only examined in vivo neuronal activity in the frontal neurons, it remains to be tested whether NMDAR co-agonists can boost neuronal activity in other brain regions in schizophrenia-like model mice. (3) It is possible that the drug doses we used are not adequate to reverse hyperactivity in schizophrenia-like model. Doses were selected based on prior studies which haven shown efficacy in schizophrenia-like models. For example, DCS at 30 mg/kg was shown to be efficacious in animals and patients [100–102], and sarcosine at the doses we used improved the schizophrenia-like phenotypes in rodents [70, 71]. It thus unlikely that dose was a major contributing factor to the lack of efficacy. (4) Our schizophrenia model was induced in the adult and has certain distinct alterations than the typical developmental or genetic model of schizophrenia (Ju et al., 2019). Thus, it remains to be tested whether these NMDAR modulators have significant impact on neural activity in developmental model of schizophrenia.

In addition to the co-agonist strategy used in this and other works, an array of direct NMDAR enhancers have been generated and tested. The more interesting and potentially useful ones are the positive allosteric modulators of NMDARs (NMDAR-PAMs) [103–105]. These PAMs include UBP compound with different subunit-selective properties [105, 106], and series of subunit-selective PAMs from Genentech [107, 108]. One of the NMDAR-PAMs, GNE-8324, showed selective potentiation of NMDAR synaptic responses in the inhibitory neurons [108]. Use of allosteric NMDAR enhancers can avoid some of the issues or uncertainty faced by NMDAR co-agonists, such as specific subcellular locations of NMDARs [109] and binding site saturation. In addition, since some PAMs show much higher potency in enhancing NMDAR responses, they may also be able to overcome the potential issue of reduced density of NMDAR or inhibitory neurons in the schizophrenia brains as discussed above. Thus, we suggest their effects in schizophrenia is worthy of testing.

## Conclusions

We failed to find significant impact of NMDAR modulators DCS and sarcosine on neural spiking in vivo in both schizophrenia-like model and wild type mice. This finding is consistent with the lack of efficacy of these modulators in some human clinical trials. We have discussed possible reasons for this strategy not to be effective in enhancing neuronal spiking and suggest that allosteric enhancers of NMADRs might avoid some of the potential issues and are worthy of testing.

## Supplementary information

**Additional file 1 Figure S1.** Effect of DCS on locomotion in the open field test. Hyperlocomotion was seen in anti-Nrg1 mice, which was not affected by injection of DCS (bar). 8 mice (Rag), 8 mice (Nrg1).

**Additional file 2 Figure S2.** Effect of DCS injection on neural activity in schizophrenia-like model mice in vivo. These data were the raw data obtained in the same experiments as those in Fig. 4. Amplitude, frequency or integrated area of spontaneous Ca^2+^ responses were shown before and after DCS injection, measured using CAG-GCaMP6s or CaMKII-GCaMP6s. A. Higher neuronal activity was observed in the anti-Nrg1 group but no effect of DCS, measured by the amplitude of Ca^2+^ responses. For CAG-GCaMP6s, basal activity, Rag vs. Nrg1, F_1, 292_ = 16.21, *P* < 0.05; with DCS, Rag vs. Nrg1, F_1, 292_ = 16.21, *P* < 0.001. For CaMKII-GCaMP6s, basal activity, Rag vs. Nrg1, F_1, 494_ = 7.163, *P* < 0.01). B. Higher neuronal activity was observed in the anti-Nrg1 group but no effect of DCS, measured by the frequency of Ca^2+^ responses. For CAG-GCaMP6s, with DCS, Rag vs. Nrg1, F_1, 292_ = 11.01, *P* < 0.01. For CaMKII-GCaMP6s, basal activity, Rag vs. Nrg1, F_1, 494_ = 5.22, *P* < 0.01. C. Higher neuronal activity was observed in the anti-Nrg1 group but no effect of DCS, measured by the total activity of Ca^2+^ responses. For CAG-GCaMP6s, basal activity, Rag vs. Nrg1, F_1, 292_ = 22.71, *P* < 0.001; with DCS, Rag vs. Nrg1, F_1, 292_ = 22.71, *P* < 0.01. For CaMKII-GCaMP6s, basal activity, Rag vs. Nrg1, F_1, 494_ = 5.22, *P* < 0.05; with DCS, Rag vs. Nrg1, F_1, 494_ = 5.22, *P* < 0.05.

**Additional file 3 Figure S3.** Sarcosine does not affect neural activity in schizophrenia-like model mice. These data were the raw data obtained in the same experiments as those in Fig. 5. Amplitude, frequency or integrated area of spontaneous Ca^2+^ responses were shown before and after sarcosine injection, measured using Synapsin-GCaMP6s (A), or CaMKII-GCaMP6s (B). A. In neurons expressing synapsin-GCaMP6s virus, injection of 0.3 g/kg or 1 g/kg sarcosine did not significantly change the amplitude (A1), frequency (A2) or total activity (A3) of spontaneous Ca^2+^ responses, in either anti-Nrg1 or anti-Rag mice. Increased basal neural activity was observed in the anti-Nrg1 group. A1. 0.3 g/kg, basal activity, Rag vs. Nrg1, F_1, 442_ = 43.78, *P* < 0.001; with sarcosine, anti-Rag vs. anti-Nrg1, F_1, 442_ = 43.78, *P* < 0.001. A2. 0.3 g/kg, basal activity, anti-Rag vs. anti-Nrg1, F_1, 442_ = 32.48, *P* < 0.001; with sarcosine, anti-Rag vs. anti-Nrg1, F_1, 442_ = 32.48, *P* < 0.001. 1.0 g/kg, basal activity, anti-Rag vs. anti-Nrg1, F_1, 432_ = 15.62, *P* < 0.05; with sarcosine, anti-Rag vs. anti-Nrg1, F_1, 442_ = 15.62, *P* < 0.01. A3. 0.3 g/kg, basal activity, anti-Rag vs. anti-Nrg1, F_1, 442_ = 41.34, *P* < 0.001; with sarcosine, anti-Rag vs. anti-Nrg1, F_1, 442_ = 41.34, *P* < 0.001. B. In neurons expressing CaMKII-GCaMP6s virus, injection of 0.3 g/kg or 1 g/kg sarcosine did not significantly alter the amplitude (B1), frequency (B2) or total activity (B3) of Ca^2+^ responses, in either anti-Nrg1 or anti-Rag mice. Increased neuronal activity was observed in the anti-Nrg1 group. B1. 1.0 g/kg, basal activity, anti-Rag vs. anti-Nrg1, F_1, 414_ = 17.24, *P* < 0.01; with sarcosine, anti-Rag vs. anti-Nrg1, F_1, 414_ = 17.24, *P* < 0.01. B2. 0.3 g/kg, basal activity, anti-Rag vs. anti-Nrg1, F_1, 348_ = 7.054, *P* < 0.05. B3. 1.0 g/kg, basal activity, anti-Rag vs. anti-Nrg1, F_1, 414_ = 4.78, *P* < 0.05.

**Additional file 4 Figure S4.** Effect of glycine on locomotion in the open field test and in vivo neuronal spike rate. A. Anti-Nrg1 mice showed hyperlocomotion compared to anti-Rag group. Glycine injection did not significantly alter locomotion in either anti-Rag or anti Nrg1 mice. 8 mice (Rag), 8 mice (Nrg1). B. Mean spike rate showed large reduction after glycine injection in both anti-Rag and anti-Nrg1 group (paired t-test, *P* < 0.001). C. Burst spike rate was not significantly altered by glycine injection.

## Data Availability

The datasets used and/or analyzed during the current study are available from the corresponding author on reasonable request.

## Abbreviations

anti-Nrg1: Anti-Neuregulin1
anti-Rag: Anti-Ragweed
DCS: D-cycloserine
FrA: Frontal association cortex
GlyT-1: Glycine transporter 1
mPFC: Medial prefrontal cortex
NMDAR: N-methyl-D-aspartate receptor
NRG1: Neuregulin1
