# Supplementary material for: The impact of D-cycloserine and sarcosine on in vivo frontal neural activity in a schizophrenia-like model
Source: BMC Psychiatry. 2019 Oct 25;19:314. doi: 10.1186/s12888-019-2306-1 (PMC6814999; doi:10.1186/s12888-019-2306-1)

Yao et al.,Supplementary Figure 4

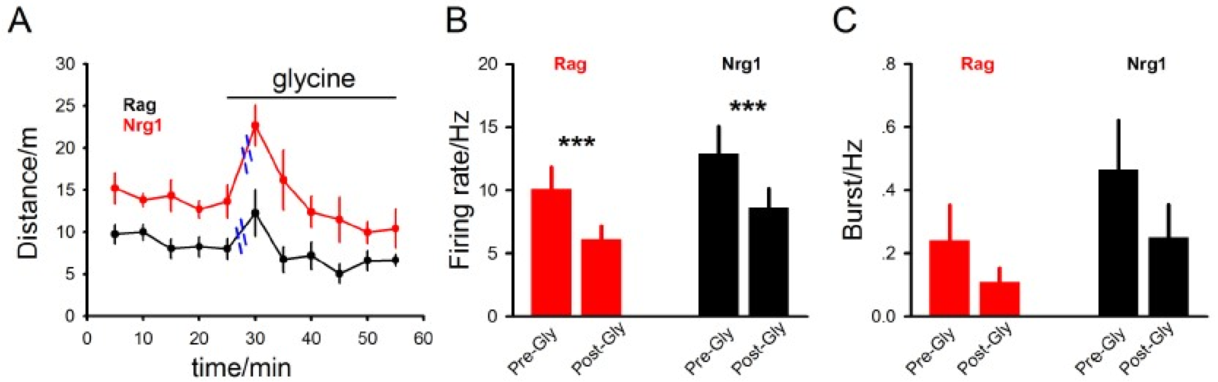

Supplement: Supplementary file 4 [file 12888_2019_2306_MOESM4_ESM.docx]
